# Worse disease-free, tumor-specific, and overall survival in surgically-resected lung adenocarcinoma patients with *ALK* rearrangement

**DOI:** 10.18632/oncotarget.20973

**Published:** 2017-09-18

**Authors:** Qiongqiong Gao, Pupu Li, Xiangli Jiang, Zhongli Zhan, Qingna Yan, Bo Zhang, Chun Huang

**Affiliations:** ^1^ Tianjin Medical University Cancer Institute & Hospital, National Clinical Research Center for Cancer, Tianjin 300060, P.R. China; ^2^ Key Laboratory of Cancer Prevention and Therapy, Tianjin 300060, P.R. China; ^3^ Tianjin’s Clinical Research Center for Cancer, Tianjin 300060, P.R. China; ^4^ Department of Thoracic Oncology, Tianjin Cancer Institute & Hospital, Tianjin 300060, P.R. China; ^5^ Department of Pathology, Tianjin Lung Cancer Center, Tianjin Cancer Institute & Hospital, Tianjin 300060, P.R. China; ^6^ Department of Ultrasound Diagnosis, Second Hospital of Tianjin Medical University, Tianjin 300060, P.R. China

**Keywords:** anaplastic lymphoma kinase rearrangement, lung adenocarcinoma, surgical resection, clinicopathological characteristics, treatment outcome

## Abstract

**Introduction:**

This study determined the prevalence of anaplastic lymphoma kinase (*ALK*) rearrangement, and identified the associations of *ALK* rearrangement with clinicopathologic characteristics and treatment outcomes in patients with surgically-resected stage I-III lung adenocarcinoma.

**Methods:**

A total of 534 surgically-resected lung adenocarcinoma patients were studied. The prevalence of ALK protein over-expression was determined by a fully-automated immunochemistry assay (with mouse monoclonal Ventana D5F3 antibody), and the associations of *ALK* rearrangement with clinicopathologic characteristics and treatment outcomes were analyzed.

**Results:**

Forty-two (7.9%) of the 534 lung adenocarcinoma patients were *ALK* IHC-positive. *ALK* rearrangement was significantly associated with younger age (*P* = 0.011), high T-stage (*P* = 0.025), high pathologic stage (*P* = 0.002), solid predominant adenocarcinoma with mucin production (*P* = 0.006), invasive mucinous adenocarcinoma (*P* = 0.009), and receipt of adjuvant therapy after surgery (*P* = 0.036), but no significant associations were found between the *ALK* rearrangement and sex or smoking status. *ALK* IHC-positivity was significantly associated with a shorter disease-free survival, tumor-specific survival, and overall survival (*P* = 0.001, 0.026, and 0.007, respectively). Multivariate analysis showed that *ALK* IHC-positivity was an adverse prognostic factor for disease-free survival (HR, 1.80; 95% CI 1.18-2.77; *P* = 0.007), tumor-specific survival (HR, 2.59; 95% CI 1.35-4.97; *P* = 0.004), and overall survival (HR, 1.92; 95% CI 1.07-3.44; *P* = 0.030).

**Conclusion:**

The clinical characteristics of patients with *ALK*-positive lung adenocarcinoma were similar to those of *EGFR*-mutated patients. *ALK* rearrangement was an adverse prognostic factor in surgically-resected lung adenocarcinoma patients.

## INTRODUCTION

Lung cancer is the leading cause of cancer-related deaths in China [1]. Globally, non-small-cell lung cancer (NSCLC) accounts for 85% of lung cancer cases [2]. Although surgical resection may be curative in some patients, it is achievable only for early-stage NSCLC, and the majority of cases are diagnosed at an advanced stage. For advanced cases, cytotoxic chemotherapy is still the first-line treatment. In the last 5 years, evidence has accumulated that there are genetically-defined molecular subsets of lung adenocarcinoma, the most remarkable subset being patients with epidermal growth factor receptor (*EGFR*) gene mutations, which defines a small subset of patients with NSCLC who have high sensitivity to *EGFR*-tyrosine kinase inhibitors (TKIs) such as gefitinib and erlotinib [3, 4].

Patients with echinoderm microtubule-associated protein-like 4 gene and anaplastic lymphoma kinase gene (*EML4-ALK*) fusion is another important subset of NSCLC. This is caused by a small inversion of chromosome 2p (the N-terminal half of *EML4* encompassing the basic region) and the hydrophobic echinoderm microtubule-associated protein-like protein (HELP) domain, and a portion of the WD-repeat region becomes fused to the intracellular juxtamembrane region of *ALK* [5]. The *EML4-ALK* fusion gene possesses powerful oncogenic activity, both *in vivo* and *in vitro* [5, 6]. It has been reported that there are 10 or more subtypes of the *EML4-ALK* fusion gene, with the E13:A20 and E6a/b:A20 types being the most common ones (incidence rates, 33% and 29%, respectively) [7, 8]. Recently, researchers have identified other *ALK* fusion partners in addition to *EML4*, including TRK-fused gene (*TFG*) [9], kinesin family member 5B (*KIF5B*) [10], kinesin light chain 1 (*KLC1*) [11], huntingtin-interacting protein 1 (*HIP1*) [12], translocated promoter region (*TPR*) [13], and *SEC31A* [14].

Methods of detecting *ALK* gene fusion include immunohistochemistry (IHC), reverse transcriptase-polymerase chain reaction (RT-PCR) technology, and fluorescence *in situ* hybridization (FISH). In 2013, the National Comprehensive Cancer Network (NCCN) stated that FISH was the ‘gold standard’ method to detect *ALK* fusion genes. However, FISH is expensive and it is difficult to determine the overall tumor morphology and heterogeneity with its use [15], while RT-PCR requires high-quality primers and more RNA [16]. In contrast, IHC is economical, practical, and efficient, and this method is now widely used in routine pathology laboratory testing. However, there is some subjectivity in evaluating staining results in IHC, and the accuracy of the method depends largely on the quality of the antibodies used [17]. Therefore, antibodies with high specificity and sensitivity are an important requirement. A recent study that compared 4 different *ALK* antibodies - D5F3 (Ventana), D5F3 (CST), 1A4/1H7 (OriGene Tech), and 5A4 (Abcam) - reported that their sensitivities were 93.8%, 84.4%, 93.8%, and 56.3%, respectively [18]. Notably, the newly developed Ventana monoclonal antibody (D5F3) has greatly improved the specificity and sensitivity of IHC testing [19], and one study has suggested that it can be used as a stand-alone test in cases displaying an unequivocal staining pattern [20]. Recently, based on a fully automated IHC assay developed by Ventana Medical Systems, the Ventana ALK (D5F3) IHC kit was approved to detect *ALK* fusion genes by the US Food and Drug Administration (FDA). The sensitivity and specificity of this IHC assay have been reported to be 100% and 98%, respectively [21].

The prevalence of *ALK* rearrangement in patients with NSCLC has been found to range from 1.4% to 13% [22–25], and to be most common in those with a young age, a never or light smoking history, an abundant signet ring cell or solid pattern histology, and wild-type *EGFR* or *KRAS* gene mutations [22–32]. The incidence rate of *ALK* rearrangement in NSCLC with wild-type *EGFR* or *KRAS* gene mutations has been reported to range from 25.7% to 34% [22, 29, 33]. Although crizotinib, a small-molecular TKI, is now approved for the treatment of advanced *ALK*-positive NSCLC in view of its favorable therapeutic effect and safety in clinical trials [34, 35], the prognostic influence of *ALK* rearrangement in early-stage NSCLC in the absence of crizotinib treatment remains unclear.

The aim of the present study was to detect over-expression of ALK protein with the Ventana IHC test and to examine the associations of *ALK* rearrangement with clinicopathologic characteristics and treatment outcomes in patients with early-stage lung adenocarcinoma.

## RESULTS

### Prevalence and clinicopathologic characteristics of patients harboring *ALK* rearrangement

Data on a total of 534 completely-resected lung adenocarcinoma patients were analyzed. The Ventana IHC test for *ALK* rearrangement was performed in all patients. Forty-two (7.9%) of the 534 patients were IHC-positive for *ALK*.

The clinicopathologic characteristics of the 534 patients are summarized in Table 1. Two hundred and sixty-one patients (48.8%) were male, and 273 (51.2%) were female; 302 (56.6%) who had smoked less than 100 cigarettes in their lifetime were classified as never-smokers, and 232 (43.4%) were classified as were smokers. Tumor size (cm) ranged from 0.2 to 12. The pathologic stage was I in 309 patients (57.9%), II in 53 (9.9%), and III in 172 (32.2%). The histopathologic subtypes, determined according to the new IASLC/ATS/ERS classification of lung adenocarcinoma [36], were adenocarcinoma in preinvasive lesions in 17 patients (3.2%) and invasive adenocarcinoma in 481 patients (90.1%), with lepidic predominant, acinar predominant, papillary predominant, micropapillary predominant, and solid predominant with mucin production subtypes present in 111 (20.8%), 214 (40.1%), 29 (5.4%), 29 (5.4%), and 98 patients (18.4%), respectively, and variants of invasive adenocarcinoma in 36 patients (6.7%). Postoperative adjuvant chemotherapy or radiotherapy was administered to 282 patients (52.8%). *ALK* rearrangement was significantly associated with younger age (median age, 57.5 years in the *ALK* IHC-positive group vs 60 years in the *ALK* IHC-negative group; *P* = 0.011), high tumor status (pT4; *P* = 0.025), high pathologic stage (IIIB; *P* = 0.002), solid predominant adenocarcinoma with mucin production (*P* = 0.006), invasive mucinous adenocarcinoma (*P* = 0.009), and receipt of adjuvant therapy after surgery (*P* = 0.036). However, there were no significant associations with sex (*P* = 0.634), smoking status (*P* = 0.333), ECOG PS score (*P* = 0.587), tumor size (*P* = 0.955), and lymph node status (*P* = 0.131).

**Table 1:** Prevalence of *ALK* rearrangement and its association with clinicopathologic characteristics in patients with early-stage lung adenocarcinoma

	ALK D5F3 (Ventana) IHC				*P*-value
	Positive		Negative		
	No. of patients	%	No. of patients	%	
Total	42	7.9	492	92.1	
Sex:					
Male	19	45.2	242	49.2	0.634
Female	23	54.8	250	50.8	
Age at surgery, years:					
Median	57.5		60		0.011^a^
Range	41-72		35-79		
Smoking status:					
Never-smoker	27	64.3	275	55.9	0.333
Smoker	15	35.7	217	44.1	
ECOG PS:					
0	13	31.0	131	26.6	0.587
1	29	69.0	361	73.4	
Family history of cancer:					
No	34	81.0	410	83.3	0.669
Yes	8	19.0	82	16.7	
Localization of primary tumor:					
Upper right lobe	5	11.9	155	31.5	0.010
Middle right lobe	5	11.9	47	9.6	
Lower right lobe	13	31.0	96	19.5	
Upper left lobe	6	14.3	109	22.2	
Lower left lobe	10	23.8	72	14.6	
Dragging in several lobes	3	7.1	13	2.6	
Operating methods:					
*Anatomy:*					
Wedge resection	1	2.4	8	1.6	0.103
Sleeve resection	1	2.4	4	0.8	
Segmentectomy	0	0	7	1.4	
Lobectomy	31	73.8	421	85.6	
Bilobectomy	5	11.9	25	5.1	
Pneumonectomy	2	4.8	8	1.6	
Others	2	4.8	20	4.1	
*Technique:*					
Open thoracotomy	29	69.0	350	71.1	0.860
Thoracoscopy	13	31.0	142	28.9	
Tumor size, cm:					
Median	3		3		0.955^a^
Range	0.5-12		0.2-12		
Tumor status:					
T1	21	50.0	295	60.0	0.102
T2	13	31.0	139	28.3	
T3	1	2.4	26	5.3	
T4	7	16.7	32	6.5	
Lymph node status:					
N0	22	52.4	320	65.0	0.034
N1	2	4.8	37	7.5	
N2	16	38.1	132	26.8	
N3	2	4.8	3	0.6	
Stage:					
IA	11	26.2	226	45.9	0.008
IB	7	16.7	65	13.2	
IIA	5	11.9	40	8.1	
IIB	0	0	8	1.6	
IIIA	11	26.2	129	26.2	
IIIB	8	19.0	24	4.9	
Histopathologic subtypes:					
*Preinvasive lesions*:					0.006
Atypical adenocarcinoma hyperplasia	0	0	1	0.2	
Adenocarcinoma *in situ*	1	2.4	5	1.0	
Minimally invasive adenocarcinoma	0	0	10	2.0	
*Invasive adenocarcinoma*:					
Lepidic predominant	4	9.5	107	21.7	
Acinar predominant	9	21.4	205	41.7	
Papillary predominant	3	7.1	26	5.3	
Micropapillary predominant	3	7.1	26	5.3	
Solid predominant with mucin production	15	35.7	83	16.9	0.006^b^
*Variants of invasive adenocarcinoma:*					
Invasive mucinous adenocarcinoma	7	16.7	25	5.1	0.009^c^
Colloid	0	0	2	0.4	
Enteric	0	0	2	0.4	
Adjuvant therapy:					
No	13	31.0	239	48.6	0.036
Yes	29	69.0	253	51.4	

^a^ Wilcoxon rank-sum test.

^b^ Comparison between solid predominant with mucin production and other types.

^c^ Comparison between invasive mucinous adenocarcinoma and other types.

*ALK*, anaplastic lymphoma kinase; *ECOG PS*, Eastern Cooperative Oncology Group performance status; *IHC*, immunohistochemistry.

### Treatment outcomes

The median follow-up duration for the patients studied was 29.0 months. At the time of analysis, 451 patients (84.3%) were still alive, with 287 (92.9%) in pathologic stage I, 41 (77.4%) in pathologic stage II, and 123 (71.5%) in pathologic stage III. However, 83 (15.7%) of the 534 patients had died, 75 (90.4%) of whom had tumor-related deaths, while 8 (9.6%) had tumor-unrelated deaths. Forty-one (49.4%) of these patients were male and 42 (50.6%) were female, while 47 (56.6%) were never-smokers and 36 (43.4%) were smokers. The pathologic stage was I in 22 (26.5%) of the 83 patients, II in 12 patients (14.5%), and III in 49 (59.0%). The histopathologic subtypes were adenocarcinoma with atypical adenocarcinoma hyperplasia in 1 (1.2%), lepidic predominant in 14 (16.9%), acinar predominant in 30 (36.1%), papillary predominant in 3 (3.6%), micropapillary predominant in 4 (4.8%), solid predominant with mucin production in 23 (27.7%), invasive mucinous adenocarcinoma in 6 (7.2%), and colloid in 2 (2.5%). Fourteen patients (16.9%) were *ALK* IHC-positive, while 69 (83.1%) were *ALK* IHC-negative.

Mean overall survival was significantly shorter in *ALK* IHC-positive patients than in the *ALK* IHC-negative group (46.0 months vs 57.5 months, respectively; *P* = 0.007). In addition, the overall mortality rate was significantly higher in the *ALK* IHC-positive group than in the *ALK* IHC-negative group (33.3% vs 14.0%, respectively; *P* = 0.003), and the 2-year overall survival rate after surgery was significantly lower (78.5% vs 90.2%, respectively; *P* = 0.032). Moreover, mean tumor-specific survival was significantly shorter in the *ALK* IHC-positive group than in the *ALK* IHC-negative group (47.8 months vs 58.1 months, respectively; *P* = 0.026), and the 2-year tumor-specific survival rate of *ALK* IHC-positive patients was lower than that of *ALK* IHC-negative patients (82.7% vs 91.3%, respectively; *P* = 0.092).

Tumor recurrences occurred in a total of 199 patients (37.3%), 25 of whom (12.6%) were in the *ALK* IHC-positive group and 174 (87.4%) in the *ALK* IHC-negative group. The mean disease-free survival was 39.0 months in *ALK* IHC-negative patients versus 26.3 months in *ALK* IHC-positive patients (*P* = 0.001), and the tumor recurrence rate was significantly higher in the *ALK* IHC-positive group (59.5% vs 35.4%, respectively; *P* = 0.003).

**Figure 1:**
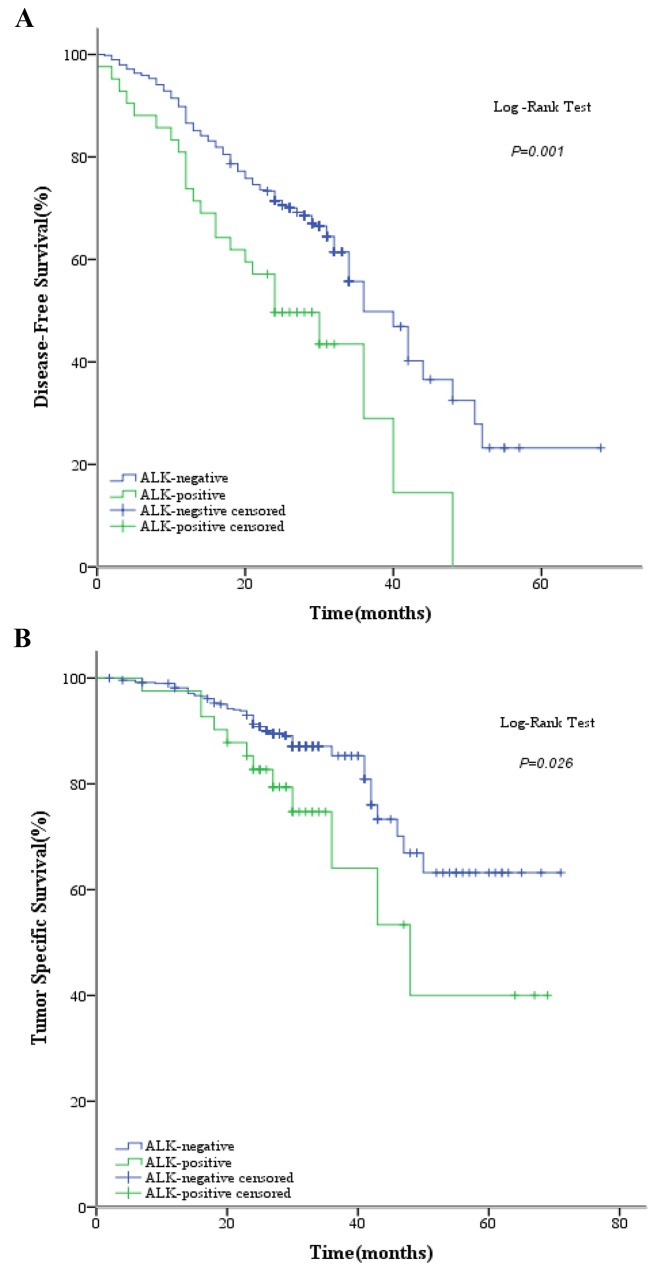

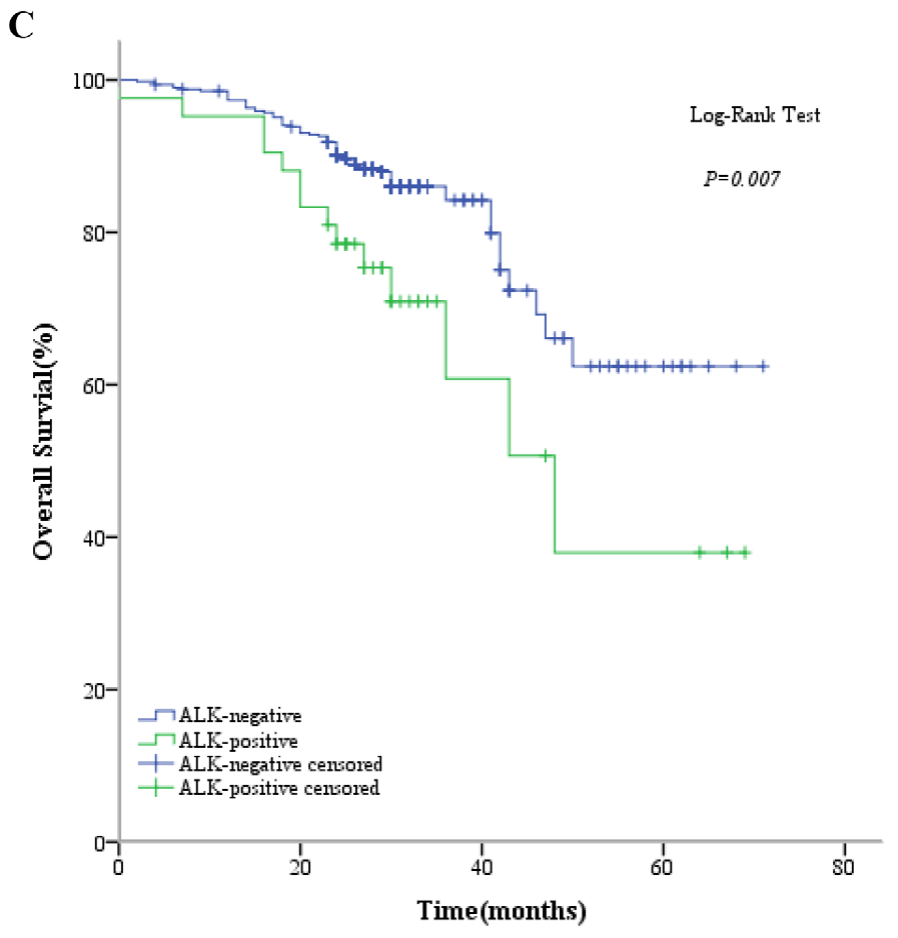
Kaplan-Meier curves showing **(A)** disease-free survival (DFS) by ALK D5F3 (Ventana) IHC; **(B)** tumor-specific survival (TSS) by ALK D5F3 (Ventana) IHC; and **(C)** overall survival (OS) by ALK D5F3 (Ventana) IHC.

Clinical outcomes, including disease-free survival (DFS), tumor-specific survival (TSS), and overall survival (OS) in the 2 patient groups are shown in Figure 1, and the clinicopathologic characteristics of the 199 patients with recurrences are shown in Table 2. The median age of the *ALK* IHC-positive group was significantly lower than that of the *ALK* IHC-negative group (53 years vs 60 years, respectively; *P* = 0.002). However, there were no significant differences for other clinicopathologic characteristics, such as sex (*P* = 0.391), smoking status (*P* = 0.513), tumor status (*P* = 0.700), lymph node status (*P* = 0.061), histopathologic subtypes (*P* = 0.070), or adjuvant therapy (*P* = 0.266). In addition, there were no statistically significant differences between the 2 groups for the first recurrence site (*P* = 0.865) and the number of recurrence sites (*P* = 0.328) [Table 2].

**Table 2:** Clinicopathologic characteristics of 199 patients with recurrent lung adenocarcinoma

	ALK D5F3 (Ventana) IHC				*P*-value
	Positive		Negative		
	No. of patients	%	No. of patients	%	
Tumor recurrence	25	59.5	174	35.4	0.003
Sex:					
Male	9	36.0	82	47.1	0.391
Female	16	64.0	92	52.9	
Age at surgery, years:					
Median	53		60		0.002^a^
Range	41-71		35-77		
Smoking status:					
Never-smoker	17	68.0	102	58.6	0.513
Smoker	8	32.0	72	41.4	
ECOG PS:					
0	9	36.0	44	25.3	0.332
1	16	64.0	130	74.7	
Family history of cancer:					
No	18	72.0	144	82.8	0.268
Yes	7	28.0	30	17.2	
Localization of primary tumor:					
Upper right lobe	4	16.0	47	27.0	0.171
Middle right lobe	1	4.0	16	9.2	
Lower right lobe	8	32.0	39	22.4	
Upper left lobe	3	12.0	39	22.4	
Lower left lobe	8	32.0	24	13.8	
Dragging in several lobes	1	4.0	9	5.2	
Operating methods:					
*Anatomy:*					
Wedge resection	0	0.0	2	1.1	0.769
Sleeve resection	0	0.0	3	1.7	
Segmentectomy	0	0.0	1	0.6	
Lobectomy	21	84.0	136	78.2	
Bilobectomy	1	4.0	14	8.0	
Pneumonectomy	2	8.0	5	2.9	
Others	1	4.0	13	7.5	
*Technique:*					
Open thoracotomy	17	68.0	126	72.4	0.640
Thoracoscopy	8	32.0	48	27.6	
Tumor size, cm:					
Median	3		3		0.690^a^
Range	0.7-12		0.3-12		
Tumor status:					
T1	12	48.0	83	47.7	0.700
T2	7	28.0	57	32.8	
T3	1	4.0	13	7.5	
T4	5	20.0	21	12.1	
Lymph node status:					
N0	8	32.0	77	44.3	0.061
N1	1	4.0	14	8.0	
N2	14	56.0	82	47.1	
N3	2	8.0	1	0.6	
Stage:					
IA	4	16.0	51	29.3	0.118
IB	3	12.0	11	6.3	
IIA	2	8.0	17	9.8	
IIB	0	0.0	4	2.3	
IIIA	9	36.0	74	42.5	
IIIB	7	28.0	17	9.8	
Histopathologic subtypes:					
*Preinvasive lesions*:					0.070
Adenocarcinoma *in situ*	1	4.0	3	1.7	
Minimally invasive adenocarcinoma	0	0.0	1	0.6	
*Invasive adenocarcinoma*:					
Lepidic predominant	2	8.0	29	16.7	
Acinar predominant	5	20.0	70	40.2	
Papillary predominant	1	4.0	6	3.4	
Micropapillary predominant	3	12.0	17	9.8	
Solid predominant with mucin production	8	32.0	38	21.8	
*Variants of invasive adenocarcinoma:*					
Invasive mucinous adenocarcinoma	5	20.0	7	4.0	
Colloid	0	0.0	2	1.1	
Enteric	0	0.0	1	0.6	
Adjuvant therapy:					
No	6	24.0	64	36.8	0.266
Yes	19	76.0	110	63.2	
First site of recurrence:					
Lung	9	36.0	58	33.3	0.865
Bone	4	16.0	33	19.0	
Brain	5	20.0	29	16.7	
Pleura	1	4.0	17	9.8	
Liver	1	4.0	14	8.0	
Others	5	20.0	23	13.2	
Number of recurrence sites:					
Single site	20	80.0	154	88.5	0.328
Multiple sites	5	20.0	20	11.5	

^a^ Wilcoxon rank-sum test.

*ALK*, anaplastic lymphoma kinase; *ECOG PS*, Eastern Cooperative Oncology Group performance status; *IHC*, immunohistochemistry.

### Prognostic value of *ALK* rearrangement in surgically-resected lung adenocarcinoma patients

As shown in Table 3, univariate analysis indicated that disease-free, tumor-specific, and overall survival were significantly shorter in patients with a large tumor size, high tumor status, lymph node involvement, and solid predominant with mucin production adenocarcinoma. Adjuvant therapy was significantly associated with DFS (HR [hazard ratio], 1.93; 95% CI 1.44-2.59; *P* < 0.001) and TSS (HR, 0.17; 95% CI 0.09-0.33; *P* < 0.001). *ALK* IHC-positivity was significantly associated with a shorter DFS (HR, 1.98; 95% CI 1.30-3.01; *P* = 0.001), TSS (HR, 2.00; 95% CI 1.07-3.73; *P* = 0.029), and OS (HR, 2.16; 95% CI 1.21-3.85; *P* = 0.009).

**Table 3:** Disease-free, tumor-specific, and overall survival analyses for the 534 surgically-resected lung adenocarcinoma patients

	No. of recurrences	HR for recurrence (95% CI)				No. of tumor related deaths	HR for tumor-related death (95% CI)				No. of deaths	HR for death (95% CI)			
		Univariate	*P*-value	Multivariate	*P*-value		Univariate	*P*-value	Multivariate	*P*-value		Univariate	*P*-value	Multivariate	*P*-value
Sex:															
Male	91	1000 (ref)				36	1000 (ref)				41	1000 (ref)			
Female	108	1.07 (0.81-1.41)	0.650			39	0.92 (0.58-1.45)	0.720			42	0.88 (0.57-1.36)	0.561		
Age at surgery, years		1000 (ref)					1000 (ref)					1000 (ref)			
	199	0.85 (0.64-1.12)	0.237			75	0.99 (0.97-1.02)	0.650			83	1.11 (0.72-1.71)	0.652		
Smoking status:															
Never smoker	119	1000 (ref)				45	1000 (ref)				47	1000 (ref)			
Smoker	80	0.93 (0.70-1.24)	0.632			30	1.03 (0.64-1.63)	0.917			36	1.16 (0.75-1.80)	0.496		
ECOG PS:															
0	53	1000 (ref)				22	1000 (ref)				24	1000 (ref)			
1	146	1.09 (0.79-1.49)	0.599			53	0.94 (0.57-1.54)	0.797			59	0.95 (0.59-1.53)	0.837		
Technique:															
Open thoracotomy	143	1000 (ref)				56	1000 (ref)				62	1000 (ref)			
Thorascopy	56	0.85 (0.62-1.16)	0.293			19	0.73 (0.43-1.23)	0.235			21	0.74 (0.45-1.21)	0.224		
Tumor size, cm:		1000 (ref)					1000 (ref)					1000 (ref)			
	199	1.41 (1.07-1.88)	0.016	1.18 (0.88-1.59)	0.266	75	1.23 (1.11-1.36)	<0.001	1.17 (1.04-1.32)	0.012	83	1.79 (1.14-2.81)	0.012	1.48 (0.94-2.35)	0.094
Tumor status:															
T1-2	159	1000 (ref)				57	1000 (ref)				64	1000 (ref)			
T3-4	40	2.39 (1.69-3.39)	<0.001	1.55 (1.07-2.24)	0.020	18	2.67 (1.57-4.54)	<0.001	2.49 (1.35-4.58)	0.003	19	2.47 (1.48-4.14)	0.001	1.62 (0.94-2.79)	0.082
Lymph node status:															
Negative	85	1000 (ref)				30	1000 (ref)				33	1000 (ref)			
Positive	114	3.00 (2.26-3.98)	<0.001	2.53 (1.83-3.52)	<0.001	45	2.78 (1.75-4.42)	<0.001	4.86 (2.93-8.04)	<0.001	50	2.82 (1.81-4.38)	<0.001	2.35 (1.48-3.75)	<0.001
Adenocarcinoma subtypes:															
Others	153	1000 (ref)				54	1000 (ref)				60	1000 (ref)			
Solid predominant with mucin production	46	1.60 (1.15-2.23)	0.006	1.35 (0.97-1.89)	0.080	21	2.12 (1.27-3.53)	0.004	1.82 (1.07-3.07)	0.026	23	2.06 (1.27-3.34)	0.004	1.77 (1.08-2.90)	0.023
Adjuvant therapy:															
No	70	1000 (ref)				65	1000 (ref)				31	1000 (ref)			
Yes	129	1.93 (1.44-2.59)	<0.001	1.08 (0.76-1.52)	0.679	10	0.17 (0.09-0.33)	<0.001	0.06 (0.03-0.12)	<0.001	52	1.50 (0.96-2.33)	0.077		
*ALK* rearrangement:															
Negative	174	1000 (ref)				63	1000 (ref)				69	1000 (ref)			
Positive	25	1.98 (1.30-3.01)	0.001	1.80 (1.18-2.77)	0.007	12	2.00 (1.07-3.73)	0.029	2.59 (1.35-4.97)	0.004	14	2.16 (1.21-3.85)	0.009	1.92 (1.07-3.44)	0.030

*ALK*, anaplastic lymphoma kinase; *CI*, confidence interval; *ECOG PS*, Eastern Cooperative Oncology Group performance status; *HR*, hazard ratio.

Multivariate analysis using a Cox proportional hazards model compared the DFS, TSS, and OS of all patients. After adjusting for pathologic nodal staging, tumor staging, tumor size, and adenocarcinoma subtypes, the variables that remained significantly associated with a shorter DFS were high tumor status (HR,1.55; 95% CI 1.07-2.24; *P* = 0.020) and lymph node involvement (HR, 2.53; 95% CI 1.83-3.52; *P* < 0.001). A shorter tumor-specific survival was significantly associated with a large tumor size (HR, 1.17; 95% CI 1.04-1.32; *P* = 0.012), high tumor status (HR, 2.49; 95% CI 1.35-4.58; *P* = 0.003), lymph node involvement (HR, 4.86; 95% CI 2.93-8.04; *P* < 0.001), solid predominant with mucin production adenocarcinoma (HR, 1.82; 95% CI 1.07-3.07; *P* = 0.026), and no adjuvant therapy (HR, 0.06; 95% CI 0.03-0.12; *P* <.001). In addition, lymph node involvement (HR, 2.35; 95% CI 1.48-3.75; *P* < 0.001) and solid predominant with mucin production adenocarcinoma (HR, 1.77; 95% CI 1.08-2.90; *P* = 0.023) were adverse prognostic factors for OS. Multivariate analysis showed that *ALK* IHC-positivity was significantly associated with a shorter DFS (HR, 1.80; 95% CI 1.18-2.77; *P* = 0.007), TSS (HR, 2.59; 95% CI 1.35-4.97; *P* = 0.004), and OS (HR, 1.92; 95% CI 1.07-3.44; *P* = 0.030). These results indicate that *ALK* rearrangement is an independent adverse prognostic factor in surgically-resected lung adenocarcinoma patients.

We further analyzed factors associated with recurrences in *ALK* IHC-positive and *ALK* IHC-negative patients. As shown in Table 4, univariate analysis indicated that lymph node involvement (HR, 3.56; 95% CI 1.51-8.38; *P* = 0.004) was an adverse prognostic factor for tumor recurrences in the *ALK* IHC-positive group, and a large tumor size (HR, 1.17; 95% CI 1.08-1.27; *P* < 0.001), high tumor status (HR, 2.33; 95% CI 1.60-3.40; *P* < 0.001), lymph node involvement (HR, 2.84; 95% CI 2.10-3.83; *P* < 0.001), solid predominant with mucin production adenocarcinoma (HR, 1.63; 95% CI 1.13-2.34; *P* = 0.008), and adjuvant therapy (HR, 1.89; 95% CI 1.39-2.57; *P* < 0.001) were adverse prognostic factors for tumor recurrences in the *ALK* IHC-negative group. With multivariate analysis, lymph node involvement was found to be an independent adverse prognostic factor for disease-free survival in *ALK* IHC-negative patients (HR, 2.32; 95% CI 1.64-3.29; *P* < 0.001).

**Table 4:** Disease-free survival analysis for the *ALK* IHC-positive and *ALK* IHC-negative resected lung adenocarcinoma patients

	*ALK* IHC-positive			*ALK* IHC-negative				
	No. of recurrences	HR for recurrence (95% CI)		No. of recurrences	HR for recurrence (95% CI)			
		Univariate	*P*-value		Univariate	*P*-value	Multivariate	*P*-value
Sex:								
Male	9	1000 (ref)		82	1000 (ref)			
Female	16	1.66 (0.71-3.89)	0.241	92	0.99 (0.73-1.33)	0.944		
Age at surgery, years		1000 (ref)			1000 (ref)			
	25	1.01 (0.95-1.09)	0.688	174	1.00 (0.98-1.02)	0.769		
Smoking status:								
Never smoker	17	1000 (ref)		102	1000 (ref)			
Smoker	8	0.77 (0.32-1.87)	0.563	72	0.99 (0.73-1.34)	0.944		
ECOG PS:								
0	9	1000 (ref)		44	1000 (ref)			
1	16	1.18 (0.48-2.91)	0.714	130	1.16 (0.82-1.63)	0.401		
Technique:								
Open thoracotomy	29	1000 (ref)		126	1000 (ref)			
Thorascopy	13	0.85 (0.36-2.00)	0.708	48	0.83 (0.59-1.16)	0.269		
Tumor size, cm:		1000 (ref)			1000 (ref)		1000 (ref)	
	25	1.06 (0.86-1.30)	0.619	174	1.17 (1.08-1.27)	<0.001	1.05 (0.96-1.14)	0.316
Tumor status:								
T1-2	19	1000 (ref)		140	1000 (ref)		1000 (ref)	
T3-4	6	2.60 (0.99-6.81)	0.051	34	2.33 (1.60-3.40)	<0.001	1.47 (0.96-2.27)	0.079
Lymph node status:								
Negative	8	1000 (ref)		77	1000 (ref)		1000 (ref)	
Positive	17	3.56 (1.51-8.38)	0.004	97	2.84 (2.10-3.83)	<0.001	2.32 (1.64-3.29)	<0.001
Adenocarcinoma subtypes:								
Others	17	1000 (ref)		136	1000 (ref)		1000 (ref)	
Solid predominant with mucin production	8	1.15 (0.48-2.74)	0.758	38	1.63 (1.13-2.34)	0.008	1.38 (0.95-2.01)	0.089
Adjuvant therapy:								
No	6	1000 (ref)		64	1000 (ref)		1000 (ref)	
Yes	19	1.54 (0.61-3.92)	0.365	110	1.89 (1.39-2.57)	<0.001	1.13 (0.79-1.62)	0.494

*ALK*, anaplastic lymphoma kinase; *CI*, confidence interval; *ECOG PS*, Eastern Cooperative Oncology Group performance status; *HR*, hazard ratio.

## DISCUSSION

Previous studies have shown that the prevalence of *ALK* rearrangement in early-stage NSCLC ranges from 2.4% to 8.6%, depending on the detection method used and the population studied [28–32]. We found that the prevalence was 7.9%, which is consistent with other studies. *ALK* rearrangement has previously been shown to be more common in light smokers or never-smokers [22–30], females [23–25, 28], the solid predominant with mucin production adenocarcinoma subtype [37–39], and young patients [22, 24, 26, 28, 30, 31]. However, *ALK* rearrangement has been reported in males (*P* = 0.039) [22] and some studies have observed no sex difference [23, 26, 29–31]. Furthermore, one study concluded that *ALK* rearrangement is not related to smoking status [31]. A recent meta-analysis [40] showed that the prevalence of *ALK* rearrangement in stage III-IV NSCLC is higher than that in stages I-II, indicating that *ALK* rearrangement is more common with higher pathologic stages, which is consistent with our results (IIIB, *P* = 0.002). Although a previous study [28] showed that *ALK*-positive lung cancers have unique biologic features with early nodal metastasis despite a small-sized primary tumor (pT1, *P* = 0.02), we found that *ALK* IHC-positive lung adenocarcinoma is associated with a higher tumor stage (pT4, *P* = 0.025). As factors such as race, population, detection method, and the subsequent statistical analysis were not the same in the above studies, this may be the cause of the reported differences. Further larger-scale studies are necessary to resolve these differences.

The univariate analysis for disease-free survival in our study showed that patients receiving adjuvant therapy had a higher risk of disease recurrence (*P* < 0.001). However, this does not mean that they would not benefit from adjuvant therapy. Among the 199 patients with recurrences in our study population, 129 (64.8%) had received adjuvant therapy. Of these 129 patients, 25 (19.4%) were in pathologic stage I, and 104 (80.6%) were in pathologic stages II and III. As the use of adjuvant therapy is stage-dependent, patients with a high pathological stage comprised a high proportion (80.6%) of all patients who received adjuvant therapy. These patients are known to have a poor prognosis.

There is controversy as to whether *ALK* rearrangement is associated with the prognosis of patients with early-stage NSCLC. A retrospective study from South Korea [29] reported that *ALK* rearrangement was not associated with overall survival in lung adenocarcinoma patients who were never-smokers (*P* = 0.720), but disease-free survival was shorter in *ALK*-positive patients (*P* = 0.022). Another study, also from South Korea [28], concluded that *ALK* rearrangement was not a prognostic factor in early-stage NSCLC patients, and a study conducted in Japan [32] came to the same conclusion. The populations of these studies were all Asian. In comparison, a study from European Thoracic Oncology Platform Lungscape Project [30] found that *ALK-*positivity was related to an improved overall survival in early-stage lung adenocarcinoma patients. However, we found that *ALK* rearrangement was associated with worse disease-free survival, tumor-specific survival, and overall survival in surgically-resected lung adenocarcinoma patients. Many studies have shown that the prognosis of patients with solid predominant with mucin production adenocarcinoma is poor [41–46]. In our study, 15 (35.7%) of the 42 *ALK* IHC-positive patients had solid predominant with mucin production adenocarcinoma, and analysis of clinicopathologic characteristics showed that *ALK* rearrangement was significantly more common in patients with solid predominant with mucin production adenocarcinoma than in patients with other subtypes (*P* = 0.006). In addition, univariate analysis showed that solid predominant with mucin production adenocarcinoma was associated with a shorter DFS, TSS, and OS, and multivariate analysis showed that this subtype was an adverse prognostic factors for TSS (HR, 1.82; 95% CI 1.07-3.07; *P* = 0.026) and OS (HR, 1.77; 95% CI 1.08-2.90; *P* = 0.020). Therefore, whether the poor prognosis of *ALK*-positive patients is associated with the histopathologic subtype is uncertain.

To our knowledge, this is the largest cohort of patients in which *ALK* rearrangements have been evaluated by a fully-automated immunochemistry assay in patients with early-stage lung adenocarcinoma, unlike most other *ALK*-related studies focusing on late-stage patients. We concluded that *ALK-*positivity is correlated with worse disease-free, tumor-specific, and overall survival. In addition, this is the first study to conduct a detailed clinicopathologic analysis of Chinese patients, and its findings complement existing information on the influence of *ALK* rearrangement on the prognosis of patients with early-stage NSCLC. However, like most retrospective studies of rare genes, our study has some limitations including observational assessment of tumor recurrence, unknown *EGFR* or *KRAS* status, and a short follow-up time, Therefore, in a future study, we will obtain treatment information after tumor recurrences, analyze other rare genes, and also analyze the relationship of the solid predominant with mucin production and invasive mucinous adenocarcinoma subtypes with *ALK* rearrangement and treatment outcome. In addition, the follow-up duration will be extended to obtain more mature data for the overall survival analysis.

*In summary*, the prevalence of *ALK* rearrangement detected by Ventana IHC testing in Chinese patients with early-stage lung adenocarcinoma was 7.9%. *ALK* rearrangement was significantly associated with younger age, and with the solid predominant with mucin production and invasive mucinous adenocarcinoma subtypes. *ALK* rearrangement was an adverse prognostic factor in surgically-resected, early-stage lung adenocarcinoma patients.

## MATERIALS AND METHODS

### Patients and specimen characteristics

Patients with lung adenocarcinoma stages I-III who underwent complete surgical resection and detection of *ALK* rearrangement at Tianjin Medical University Cancer Institute and Hospital between January 2011 and December 2014 were studied. All patients had an adequate quality and quantity of formalin-fixed paraffin-embedded tissue sections available for analysis. Patients who did not undergo curative resection or had prior histories of cancer or neoadjuvant therapy were excluded. None of the patients in the study cohort had received *ALK*-targeted therapy before tumor recurrence. Data on the patients’ clinicopathologic characteristics were retrospectively obtained from the hospital’s medical recording system. The study was approved by the Research Ethics Committee of Tianjin Medical University Cancer Institute and Hospital, Tianjin, China.

### Clinicopathological evaluation and detection of *ALK* rearrangement

Pathologic staging of the patients’ NSCLC was based on the 7th edition of the tumor-node-metastasis (TNM) classification for lung cancer [47]. Histologic subtypes were determined according to the International Association for the Study of Lung Cancer/American Thoracic Society/European Respiratory Society (IASLC/ATS/ERS) classification of lung adenocarcinoma [36]. *ALK* rearrangement was detected by the fully-automated Ventana IHC assay using the pre-diluted Ventana anti-ALK (D5F3) rabbit monoclonal primary antibody, together with the Optiview DAB IHC detection kit and Optiview Amplification kit on the Benchmark XT stainer. Evaluation of the test results was determined by a binary scoring system (positive or negative for *ALK* status), as previously reported [21]. The patients’ *ALK* rearrangement and histopathologic types were determined by 2 experienced pathologists from Tianjin Lung Cancer Center (Z.Z. and Q.Y.) on a blinded basis.

### Assessment of clinical outcomes

Clinical outcomes were assessed by determining the patients’ overall survival (OS), defined as the time from the date of surgical resection to death from any cause; tumor-specific survival (TSS), defined as the time from date of surgical resection to tumor-related death; and disease-free survival (DFS), defined as the time from the date of surgical resection to disease recurrence or death from any cause. If death or recurrence was not observed in any patient, the censoring date was the last day of follow-up.

### Statistical analysis

The prevalence of *ALK* rearrangement was determined by Ventana IHC testing, and the associations of *ALK* rearrangement with the patients’ clinicopathologic characteristics and treatment outcomes were analyzed using Fisher’s exact test (for categorical data) or the Wilcoxon rank-sum test (for continuous data). The DFS, TSS, and OS were plotted using the Kaplan-Meier method and compared by a log-rank test. Multivariate analysis of treatment outcomes was performed using Cox’s proportional hazards model. All *P-*values were based on a 2-sided hypothesis. *P*-values less than 0.05 were considered statistically significant. All analyses were performed using SPSS^®^, version 20.0 (SPSS Inc., Chicago, IL, USA).

## Abbreviations

*ALK*: anaplastic lymphoma kinase
*ECOG PS*: Eastern Cooperative Oncology Group performance status
*EGFR*: epidermal growth factor receptor
*EML4*: echinoderm microtubule-associated protein-like 4
*HELP*: hydrophobic echinoderm microtubule-associated protein-like protein
*FISH*: fluorescence *in situ* hybridization
*HIP1*: huntingtin-interacting protein 1
*HR*: hazard ratio
*CI*: confidence interval
*IASLC/ATS/ERS*: International Association for the Study of Lung Cancer/American Thoracic Society/European Respiratory Society
*IHC*: immunohistochemistry
*KIF5B*: kinesin family member 5B
*KLC1*: kinesin light chain 1
*NCCN*: National Comprehensive Cancer Network
*NSCLC*: non-small cell lung cancer
*RT-PCR*: reverse transcriptase-polymerase chain reaction
*TKI*: tyrosine kinase inhibitor
*TFG*: TRK-fused gene
*TNM*: tumor-node-metastasis
*TPR*: translocated promoter region
